# Is biotechnology (more) acceptable when it enables a reduction in phytosanitary treatments? A European comparison of the acceptability of transgenesis and cisgenesis

**DOI:** 10.1371/journal.pone.0183213

**Published:** 2017-09-06

**Authors:** Damien Rousselière, Samira Rousselière

**Affiliations:** 1 Department of Economics, Management and Society, AGROCAMPUS OUEST, Angers, France; 2 UMR SMART LERECO, AGROCAMPUS OUEST, INRA, Angers, France; 3 Department of Management, Statistics and Communication, ONIRIS, Nantes, France; 4 LEMNA, University of Nantes, Nantes, France; University of Florida, UNITED STATES

## Abstract

Reduced pesticide use is one of the reasons given by Europeans for accepting new genetic engineering techniques. According to the advocates of these techniques, consumers are likely to embrace the application of cisgenesis to apple trees. In order to verify the acceptability of these techniques, we estimate a Bayesian multilevel structural equation model, which takes into account the multidimensional nature of acceptability and individual, national, and European effects, using data from the Eurobarometer 2010 73.1 on science. The results underline the persistence of clear differences between European countries and whilst showing considerable defiance, a relatively wider acceptability of vertical gene transfer as a means of reducing phytosanitary treatments, compared to horizontal transfer.

## Introduction

The European controversy on the acceptability of biotechnologies and notably their use in food has been developing since the 1990s [1]. The aspect of reducing phytosanitary treatment is often brought up by European consumers as the main reason that could lead to an acceptation of GM (genetically modified) foods as shown by different studies [2, 3, 4, 5, 6]. At the same time, an international team [7] has recently fully decoded the apple tree genome (*Malus domestica* L. Borkh) creating future possibilities for more advanced genetic engineering applications, notably for the development of new apple varieties using cisgenesis (intra-species gene transfer). This breakthrough has led authors such as Jacobsen and Shouten [8] to promote the potential of this technique, on the condition that the same communication errors as the ones regarding the development of GMOs (Genetically Modified Organisms) do not occur again. The gene marker for the resistance to antibiotics was traditionally implanted during genetic manipulation in order to differentiate between the cells for which genetic modification was successful and those where it failed. In the case of the cisgenic apple, whose initial form was developed by Vanblaere *et al*. [9], this gene marker will be eliminated (for example for the integration of a DNA element producing the enzymatic shear). Righetti *et al*. [10] show that these new breeding technologies can lead not only to cisgenic plants but also to marker-free transgenic plants.

Jacobsen and Shouten [8] propose to exclude organisms created using cisgenesis from the legislation applied to GMOs. The term "gene evolution" applied to cisgenesis (intraspecies vertical transfer) rather than "gene revolution" applied to transgenesis (interspecies horizontal transfer) would summarize, according to these researchers, the greatest potential acceptability for consumers of this type of biotechnology. The purpose of this article is to consider this question seriously at a time when the development of new technologies in genetic engineering is clouding the scientific debate and challenges the public regulation [11, 12, 13, 14]. For example, as described by Kuzma, genetic editing involves changing DNA sequences at targeted locations usually using site-directed nucleases (such as CRISPR-Cas9) and may be a safer process than first-generation GE techniques owing to its precision. Therefore “ironically the same (..) developers who claimed that the process of [Genetically engineering] does not matter for regulatory purposes are now arguing that changes to the engineering process justify looser regulatory scrutiny”[14].

Based on a survey regarding the opinions of Europeans concerning biotechnologies (Eurobarometer 73.1.), our objective is to highlight the differentiated acceptability of different genetic techniques (cisgenic or transgenic) in European countries. It what follows we use the terms genetic engineering, genetically modified and genetically modified organism following the Agricultural Biotechnology Glossary of the USDA (see https://www.usda.gov/topics/biotechnology/biotechnology-glossary) An organism that is generated through genetic engineering is considered to be genetically modified (GM) and the resulting entity is a genetically modified organism (GMO), a genetically engineered. A Bayesian multilevel structural equation model is estimated in order to take into account individual, national, and European effects of these techniques. The results underline the importance of a "country" effect, and despite strong opposition, a relatively wider acceptability for cisgenesis in comparison to transgenesis, as a means of reducing phytosanitary treatments. In a broad sense, phytosanitary treatments are pest controls (herbicide, fungicides and insecticides) corresponding to the main apple diseases. We don’t use the narrow sense (in exportation regulation) for which “Phytosanitary treatments are official pre-shipment or quarantine processes recognized internationally and used by National Plant Protection Organisations (NPPOs) to mitigate biosecurity risks associated with plants or plant-based products.” [15].

Original on an empirical level, our contribution is also original through the methodology developed: a multivariate methodology designed to explicitly take into account the complex nature of the data from a pan-European survey that measures attitudes (latent variables) rather than behavior [16, 17], which is clearly different from a previous analysis of the survey [18].

After a brief review of literature in order to position the terms of the debate between transgenesis and cisgenesis, and the specificity of its application in the case of the apple, we will present our data. We will then explain our Bayesian econometric strategy. Lastly, we will discuss the results obtained by comparing them to studies that have used similar or dissimilar methods in order to specify their validity.

## Literature review: Four proposals regarding the acceptability of transgenesis and cisgenesis

In agriculture, selectors are continuously working on the creation of new varieties with three main objectives: seeking greater productivity (yield, regularity, etc.), increasing tolerance to biotic and abiotic stress, and better quality products [19]. These different objectives, which can be linked to economic and commercial interests, are obtained either through traditional hybridization (conventional technique) or using genetic engineering (GM crops). Dealing with the problem of treating apple scab resulting from the fungus *Venturia Inaequalis* or potato mildew caused by a *Phytophthora infestans*, cisgenesis was defended as a new genetic engineering technique in various publications [8, 20]. Vanblaere *et al*. [9] highlights the creation of a new truly cisgenic variety of apple with the effective transfer of the target gene without the addition of foreign genes. This differentiated it from the "Arctic" apple, developed by the company Okanogan Specialty Fruits, created through the introduction of genes stopping browning of the apple when it is cut [21, 22];. The first fruits which appeared in 2014 were scab free [23]. A new variety with increased resistance to Fire Blight is now also available [24]. According to the advocates of this technique, it could potentially have greater consumer acceptability. Cisgenesis is quicker than natural selection for nonstandard target genes. It also avoids the risk of unfavorable characteristics linked to resistance genes being carried over. It would appear "less controversial" than transgenesis as the target genes are only transferred (artificially) between closely linked organisms that could be interbred using traditional breeding methods. As underlined by Molesini *et al*. [25], a distinction can therefore be made with another technique, intragenesis, for which the DNA sequence introduced can be a new combination of DNA. While in transgenesis, any genetic heritage (bacterial, animal, vegetable) can be used to obtain the desired qualities in the plant to be produced, cisgenesis excludes the integration of antibiotic resistant genes or promoters coming from foreign organisms, both of these dimensions being the main source of controversy [26]. Note however that alternative selection systems without antibiotic resistance genes are increasingly developed [10, 27, 28, 29].

Jochemsen [26] bases his argument on the meaning of the concept of “species” and on the role of genetic information in organisms to differentiate between species on an ethical level. According to this principle, cisgenesis does not transgress the identity of the species. However, it can have a negative impact on the integrity of the genome as this technique does not enable the transmitted DNA integration site to be chosen. In various reports, the European Commission has classified this type of technique amongst eight promising biotechnology techniques, whilst retaining that the cisgenic technique is particularly close to more traditional transgenic techniques and should, as a result, continue to be covered by the same regulations [30]. Thus, cisgenesis is covered by the 2001/18/CE directive that defines a GMO as "an organism, with the exception of human beings, whose genetic material has been modified in a manner that does not happen naturally by natural recombination and/or multiplication" It is therefore solely the genetic modification procedure that determines if a product is a GMO and not the result. Hence the assimilation to another technique, fastbreeding, in which transgenesis is used during the first stage in order to accelerate the selection process, but that only retains apples, which do not bear the gene introduced, at the final stage [31].

The results of surveys amongst Europeans underline their numerous concerns regarding scientific issues. Successive periods have highlighted that point, and since the 1990s there has been a growing mistrust regarding the application of genetic engineering to food [1]. Although strongly correlated, the acceptability of biotechnology differs considerably depending on the field of application [32, 33]. Gaskell *et al*. [34] have emphasized that the absence of acceptance of certain techniques is linked to the lack of perceived usefulness, which is even greater when the subject undergoes a less visible transformation, such as fruit and vegetables [32]. The fact that cisgenesis can be presented as a technique likely to reduce phytosanitary treatments tends to promote its acceptability [13]. Other desirable traits may refer to health (such as the development of hypoallergenic apples [35] or improved nutritional characteristics [36, 37]. Using mixed methods, Kronberger *et al*. [18] confirm this point while underlining that “cisgenesis is considered to be different from breeding and commonly classified as a form of genetic modification”. Finally, Delwaide *et al* [38], measuring consumers’ willingness to pay, show that European consumers may accept cisgenic food products more readily than transgenic food products.

We can thus formulate an initial research proposal:

H1. There is a greater acceptability of cisgenesis than transgenesis, while noting a strong correlation between the two.

The different studies carried out on the social acceptability of biotechnology all underline that the absence of perceived utility is one of the determining factors in the opposition to biotechnologies regardless of the field of application [1, 33]. Being interested in the environment can lead to perceiving certain genetic manipulation techniques, as long as they are presented as an extension of more traditional methods, as relatively acceptable. Consumers often mention the reduction of phytosanitary treatment as the main reason that could lead them to accept to accepting GM foods [2, 3, 4, 5, 6], even if its importance can be controversial [39]. Studying the acceptability of GM Tomatoes in USA, Loureiro & Bugbee [3] find that consumers are willing to pay the highest premium for the ‘‘enhanced flavor” attribute, followed by both the ‘‘enhanced nutritional value” and ‘‘pesticide reduction” attributes. The situation may be different for apples as fruit tree farming is highly dependent on phytosanitary treatments. The control of apple scabs, which has a considerably negative impact on the propensity of consumers to purchase apples [40], leads to 10 to 20 antifungal treatments per year. Using choice experiment survey in New Zealand, Kaye-Blake *et al*. [2] find that the value of GM apples is determined by the specific benefits that can be provided: the willingness to pay for GM apples increases with either improved flavor or fewer insecticides, but the premium is higher for the latter than for the former. Different studies underline that consumers are concerned by the pollution caused by the spread of pesticide residues into the environment. Heiman [6] argues that information on reduced pesticide use in GM crops primes at least two attributes simultaneously—health, and contribution (damage) to the environment. Consumers with a greater interest in science (or training in these areas) generally show accept biotechnologies more readily [35, 41, 42].

Two research proposals arise from this:

H2. Environmental concerns are important for the acceptability of both techniques
H3. General interest in science or biotechnology are important factors for the acceptability of both techniques.

Joly & Marris [43] underline the specific structure of the debate in each country, highlighting very different acceptability levels between countries, within the same country, and for different applications. Nayga *et al*. [44] emphasize a greater acceptability of genetically modified plants in South Korea than in the United States. This point is confirmed by meta-analysis of experimental economic studies underlining greater resistance from the European consumers than from American or Asian ones [45, 46]. European studies [1, 47] or comparative studies between countries [43] show that beyond the average European citizen there is a great diversity in national configurations. There is a convergence between European countries on the general attitude towards biotechnologies, with the caveat that recent members of the European Union show an increase in the number of citizens who are, *ex ante*, more favorable to them [48, 49]. Specifically concerning intragenesis, Lusk & Rozan [50, 51] and Rozan *et al*. [36] have shown, on the one hand, a greater acceptance of this technique when compared with other gene transfer techniques and, on the other, major differences between France and the United States on this point. Delwaide *et al*. [38] have estimated significant differences in WTP for cisgenesis and transgenesis across countries.

We can therefore propose:

H4. A considerable portion of the heterogeneity of individual preference towards cisgenesis and transgenesis is explained by taking the national aspects into consideration.

## Presentation of data: Eurobarometer 73.1

We used data from Eurobarometer 73.1, concerning the attitudes of Europeans towards science in 2010 (see Kronberger *et al*. [18] for univariate statistics). Approximately 1000 people per country were questioned using a random multiphase sampling process. The survey covers the population from 15 years of age and upwards residing in each member state of the European Union, as well as some associated countries (like Norway, Iceland, and Turkey). Thus a series of questions was asked with an initial scenario given that each individual was supposed to answer:

*Some European researchers think there are new ways of controlling common diseases in apples—things like scab and mildew. There are two new ways of doing this. Both mean that the apples could be grown with limited use of pesticides, and so pesticide residues on the apples would be minimal*.

*The first way is to introduce artificially a resistance gene from another species such as a bacterium or animal into an apple tree to make it resistant to mildew and scab (…) The second way is to artificially introduce a gene that exists naturally in wild/ crab apples which provides resistance to mildew and scab*.

An assimilation is made between the "vertical transfer of genes" and cisgenesis on the one hand, and between the "horizontal transfer of genes" and transgenesis on the other [1]. As previously indicated, this assimilation is correct on the whole. It does, however, ignore one of the aspects of transgenesis that requires the use of marker genes (not present in cisgenesis). This dimension is one of the controversial elements of transgenesis, absent from cisgenesis. Due to the way in which questions were asked, it is unlikely that Europeans use this argument for the acceptance of one technique rather than another. To simplify, we use the terms "cisgenesis" and "transgenesis". Note finally that according to recent studies [10], marker-free transgenic plants may be produced in the near future.

The Table 1 shows the different rates of agreement with proposals concerning the genetic manipulation of apple trees. The beliefs are generally expressed on a Likert scale (totally Agree, Agree vs disagree and totally disagree, and don’t know), except for two (label support for transgenesis and cisgenesis) when a dummy is used (yes vs no). Unfortunately, the model cannot handle responses with different distributions. Following Gaskell *et al*. [34], we dichotomize the responses and consider only the positive response (agree & totally agree) (vs. the negative ones). Note that our strategy is also a way to address the existence of country style response that may lead to biased analysis [52].

**Table 1:** Rates of agreement with different proposals concerning the genetic manipulation of apple trees.

**Gene transfer from other species**	**Sample (n = 15650)**	**Luxemburg (n = 250)**	**The Netherlands (n = 522)**	**Turkey (n = 472)**	**Hungary (n = 523)**
• Is a promising idea	42.4%	36.0%	60.7%	32.0%	46.8%
• Will be safe	28.6%	17.2%	42.9%	22.7%	37.1%
• Will harm the environment	43.3%	62.0%	34.7%	41.3%	35.0%
• Is fundamentally unnatural	69.3%	86.0%	73.0%	49.4%	72.7%
• Makes you feel uneasy	56.8%	71.2%	51.9%	45.6%	54.9%
• Should be encouraged	27.5%	14.0%	39.8%	19.3%	36.3%
• Is to be given a label	81.0%	88.4%	83.3%	61.9%	82.8%
**Gene transfer from the same species**	**Sample**	**Luxemburg**	**The Netherlands**	**Turkey**	**Hungary**
• Will be useful	60.5%	56.4%	76.6%	37.9%	36.3%
• Will be risky	41.4%	52.4%	22.6%	49.6%	82.8%
• Will harm the environment	31.2%	47.2%	22.4%	42.2%	71.7%
• Is fundamentally unnatural	52.2%	70.4%	55.6%	50.6%	37.7%
• Makes you feel uneasy	41.1%	53.2%	37.4%	47.7%	19.7%
• Should be encouraged	44.0%	32.0%	49.8%	23.1%	46.1%
• Is to be given a label	71.1%	85.2%	72.4%	62.9%	30.0%

It is important to underline that the titles of questions vary in part between the two techniques but our model is a way to handle this problem by providing various estimators for that (individual and national factors, determinants of each response, etc.). See section 4. We also report (in Table 1) the descriptive statistics for four countries (Luxemburg with the lowest support for transgenesis, the Netherlands with the highest support, Turkey with the lowest support for cisgenesis, and Hungary with the highest). The table gives us some outlines of the European heterogeneity. The first conclusion we can come to concerns the opposition of the majority towards these two techniques, making the people questioned "feel uneasy". Europeans emphasized the requirement for the labeling of these apples (81% of the transgenesis and 71% of cisgenesis). The second conclusion is that Europeans appear more favorable to vertical gene transfer for apple trees (a lower rate of people replying that this harms the environment, making them feel uneasy, a higher rate of people replied that it could be useful, or should be encouraged). But it seems that there is a highest variation between countries about this last technology: In Hungary 71.7% of the population think that cisgenesis will harm the environment, in comparison with a rate of 22.4% in the Netherlands, which leads to a gap of 49.7% between these two opposite positions. For transgenesis, the gap is only 27.3%. Finally we can also note that even in the country with the highest support, the agreement with the proposition that transgenesis should be encouraged is relatively low (only 39.8% for the Netherlands).

In Table 2, the correlations between the various responses are reported. Only one is not significant (between “gene transfer from other species is fundamentally unnatural” and “gene transfer from the same species will be useful”). As responses across and within each kind of gene transfer are highly correlated, a multivariate analysis taking this structure into account is required.

**Table 2:** Correlation between the various responses.

		Gene transfer from other species							Gene transfer from the same species						
		Is a promising idea	Will be safe	Will harm the environment	Is fundamentally unnatural	Makes you feel uneasy	Should be encouraged	Is to be given a label	Will be useful	Will be risky	Will harm the environment	Is fundamentally unnatural	Makes you feel uneasy	Should be encouraged	Is to be given a label
Gene transfer from other species	Is a promising idea	1.00	0.85*	-0.44*	-0.30*	-0.54*	0.87*	-0.17*	0.62*	-0.21*	-0.26*	-0.15*	-0.33*	0.52*	-0.07*
	Will be safe		1.00	-0.42*	-0.37*	-0.58*	0.85*	-0.27*	0.56*	-0.22*	-0.23*	-0.16*	-0.33*	0.52*	-0.14*
	Will harm the environment			1.00	0.69*	0.71*	-0.46*	0.38*	-0.17*	0.42*	0.59*	0.35*	0.42*	-0.19*	0.21*
	Is fundamentally unnatural				1.00	0.79*	-0.43*	0.56*	0.02	0.36*	0.36*	0.57*	0.42*	-0.09*	0.29*
	Makes you feel uneasy					1.00	-0.64*	0.51*	-0.18*	0.42*	0.46*	0.41*	0.67*	-0.25*	0.28*
	Should be encouraged						1.00	-0.27*	0.60*	-0.24*	-0.26*	-0.21*	-0.38*	0.68*	-0.14*
	Is to be given a label							1.00	0.13*	0.20*	0.14*	0.30*	0.23*	0.05*	0.77*
Gene transfer from the same species	Will be useful								1.00	-0.52*	-0.57*	-0.46*	-0.62*	0.89*	-0.26*
	Will be risky									1.00	0.82*	0.75*	0.79*	-0.54*	0.48*
	Will harm the environment										1.00	0.77*	0.81*	-0.54*	0.44*
	Is fundamentally unnatural											1.00	0.83*	-0.57*	0.58*
	Makes you feel uneasy												1.00	-0.69*	0.55*
	Should be encouraged													1.00	-0.37*
	Is to be given a label														1.00

Note: tetrachoric correlations reported,

*: significant at the .10 level.

We can therefore ask what determines this attitude and how the answers to different questions correlate. One hypothesis could be that the response observed depends not only on a vector of observed variables (socio-demographic factors, but also values and interests), but also on a general unobserved individual attitude (depending on a vector of individual determinants), and on a general unobserved attitude shared by the citizens of the same country. The last point leads us to try to measure the importance of the national aspect on individual attitudes.

## Econometric strategy: A multilevel structural equation model

Using a standard statistical model is not appropriate if the data studied clearly has a hierarchical structure, notably meaning that the intragroup correlation is statistically significant [53]. A bias in the estimation variance is created when all responses are considered independent. If, on the contrary, we carry out the analysis on groups, taking into account average values, the correlation between variables created in such a way is biased leading to the ecological error [54]. Using multilevel model is therefore a traditional approach method for Eurobarometer data [55].

Our data source also creates a major problem: contrary to experimental economics surveys, it does not directly measure behavior but rather attitudes. This creates the problem of misreporting or a measurement error because of a “social desirability bias”: participants may be led to “simply stating a principle” [56]. A solution is to estimate an econometric model with a measurement error using auxiliary data [57]. Another approach is to collect data from products that consumers have already purchased [58]. As European consumers are not able to buy real genetically modified apples, both techniques are inapplicable. We choose to take this data seriously within a *latent variable framework*. As in psychometrics, our hypothesis is that the attitude cannot be directly observed but inferred from the coherence of the answers given by the individuals [59, 60]. Therefore note that this latent variable framework does not have the usual economic interpretation (individual utility).

New multilevel factorial models [17, 61, 62, 63, 64] are appropriate to correctly deal with the multidimensionality of relationships between Europeans and biotechnologies without an excessive addition of parameters. They notably take into account the heterogeneity both at the individual level and at the group level.

A two individual and two national factors model is given bellow [16]. For an individual *i* in a country *j*, we have:

$$g\left(y_{rij}\right)=\sum_{h=1}^{H}\beta_{h,r}x_{h,ij}+\lambda_{1,r}^{\left(2\right)}\eta_{1j}^{\left(2\right)}+\lambda_{2,r}^{\left(2\right)}\eta_{2j}^{\left(2\right)}+\lambda_{1,r}^{\left(1\right)}\eta_{1ij}^{\left(1\right)}+\lambda_{2,r}^{\left(1\right)}\eta_{2ij}^{\left(1\right)}+u_{rj}+e_{rij}$$ (1)

With *g(*.*)* the probit function. As Grilli & Rampichini point out [17], the choice of the binary function often has little influence on the results, we choose the probit function by commodity, as the latent variable would be considered Gaussian (hence the link to the traditional factorial model). Therefore, we have *e*_*rij*_*~N(0*,*1)*.

*y*_*rij*_ the response with r = 1,…,14 for the 14 different responses, *x*_*h*,*ij*_ the independent variables. We choose the usual socio-demographic (age, gender, occupation, location) and attitudes (political scale, environmental, science and biotechnologies attitudes).

$\lambda_{1,r}^{\left(2\right)}$ et $\lambda_{2,r}^{\left(2\right)}$ corresponding to the loading of the response r on the two factors at the national level; $\lambda_{1,r}^{\left(1\right)}$ and $\lambda_{2,r}^{\left(1\right)}$ corresponding to the loadings of the response r on the two factors at the individual level,

$$\left[\begin{array}{c} \eta_{1j}^{\left(2\right)}\\ \eta_{2j}^{\left(2\right)} \end{array}\right]\sim MVN\left(0,\Omega_{\text{n}}^{\left(2\right)}\right)\;\text{and }\left[\begin{array}{c} \eta_{1ij}^{\left(1\right)}\\ \eta_{2ij}^{\left(1\right)} \end{array}\right]\sim MVN\left(0,\Omega_{\text{n}}^{\left(1\right)}\right)\;{\text{such that }\Omega }_{\text{n}}^{\left(2\right)}=\left[\begin{array}{c} \begin{array}{cc} \sigma_{\text{n}1}^{\left(2\right)2} & \\ \sigma_{\text{n}12}^{\left(2\right)} & \sigma_{\text{n}2}^{\left(2\right)2} \end{array} \end{array}\right],{\;\Omega }_{\text{n}}^{\left(1\right)}=\left[\begin{array}{cc} \sigma_{\text{n}1}^{\left(1\right)2} & \\ \sigma_{\text{n}12}^{\left(1\right)} & \sigma_{\text{n}2}^{\left(1\right)2} \end{array}\right]\;\text{and }u_{rj}\sim N\left(0,\sigma_{\text{ur}}^{2}\right),\;e_{rij}\sim N\left(0,\sigma_{\text{er}}^{2}\right).$$

For questions of model identifiability, reasonable hypotheses must be made: Setting the variance of factors at a certain value (normally a unit), and setting the coordinates of one of the responses on one of the factors to a certain value (normally zero). Then:

$$\Omega_{\text{n}}^{\left(2\right)}=\left[\begin{array}{c} \begin{array}{cc} 1 & \\ \sigma_{\text{n}12}^{\left(2\right)} & 1 \end{array} \end{array}\right]\;{\text{and }\Omega }_{\text{n}}^{\left(1\right)}=\left[\begin{array}{cc} 1 & \\ \sigma_{\text{n}12}^{\left(1\right)} & 1 \end{array}\right]$$.

In our example, the nullity of some coordinates arises naturally. We can group the different dependent variables together into two different factors: an attitude factor for cisgenic apples and an attitude factor for transgenic apples; both these factors can be correlated. As the factors have the same scale (with a unit variance), the loadings for the same response *r* can be compared between different factors on the same level or different levels. On the other hand, as the latent variables $y_{rij}^{*}$ have different scales, the loadings cannot be compared between responses, it is therefore necessary to standardize them.

We can establish an *ICC* (residual (or conditional) intraclass correlation coefficient) for the response *r* corresponding to the variance explained by the country level:

$$ICC_{r}=\frac{Var_{2}\left(y_{rij}^{*}\right)}{Var\left(y_{rij}^{*}\right)}$$ (2)

Where $Var_{1}\left(y_{rij}^{*}\right)$ is the variance at level 1 (that of the individual) and $Var_{2}\left(y_{rij}^{*}\right)$ is the variance at level 2 (country level)

This coefficient gives the percentage of the variance in the acceptance taken into consideration by the inclusion of a level.

Similarly, as for every factorial model, we can calculate the communalities, that is to say the amount of variance for the response *r* explained by the factors. The communality is also known as the variance proportion that the response *r* has in common with the other responses. The total communality is the sum of communalities of the country level ($Com_{r}^{\left(2\right)}$) and the individual level ($Com_{r}^{\left(1\right)}$).

This model is estimated within the Bayesian framework using MCMC (Markov Chain Monte Carlo) [65]. We use Realcom software developed by the Center for Multilevel Modelling [63]. This type of modeling has been shown to be unbiased for models with dichotomic or categorical response variables [66], cross-classified models [67] as well as for cases where the number of categories at the upper level is low [68, 69, 70]. The Bayesian estimator does not generally allow analytical solutions. Recourse to draws from the *posterior parameter distribution* is required. Several estimation methods are possible, the most popular being Metropolis Hastings method and Gibbs sampling. The latter is implemented in Realcom. With diffuse (or “flat”) prior parameters proposed by Browne [65], we used 100 000 iterations after an initial burn-in of 50 000 iterations. Bayesian models don’t give only one point estimate but rather an estimation of the parameter distribution. We follow Koop [71] and report the posterior means and credible interval of the parameter. The parameter can be considered as significantly different from zero if the credible intervals (at 90%, 95% or 99%) don’t include zero [72].

Lastly, the question of choice amongst all the alternative specifications arises. We follow Bayesian model selection [73] using the *DIC* (Deviance Information Criterion) proposed by Spiegelhater *et al*. [74]. A generalization of information criteria within the framework of multilevel models, *DIC*(*θ*_*i*_) is asymptomatically equivalent to the *AIC* (Akaike information criterion) in the presence of non-informative priors [74]. The weaker *DIC*(*θ*_*i*_) is, the “better” the model is. Therefore Jeffery’s rule of thumb can be used [73, 74, 75]. A difference of 10 between two DIC might definitely rule out the model with the highest DIC, as it involved that the model with lowest DIC has approximately a posterior odds of 150:1 to be the true model [73].

In Table 3, we show how our hypotheses can be tested by the parameters of our multilevel model. One of the main advantages of our empirical strategy is that we can fully *simultaneously* test the four hypotheses. Sequential testing is based on a strong assumption, namely that the hypotheses are independent from each other. This assumption is relaxed here [16].

**Table 3:** Hypotheses.

Hypothesis	Validation
H1. There is a greater acceptability of cisgenesis than transgenesis, whilst noting a strong correlation between the two.	Magnitude and Significance of the correlation between the factors at the individual level ($\sigma_{\text{n}12}^{\left(2\right)}$) and/or the national level ($\sigma_{\text{n}12}^{\left(1\right)}$)
H2. Environmental preoccupations are important for the acceptability of both techniques	Magnitude of Marginal effects of interestenvir
H3. Interests for science or biotechnology in general are factors influencing the acceptability of both techniques.	Magnitude of Marginal effects of interestscience and interestbiotech
H4. A considerable portion of the heterogeneity of individual preference towards cisgenesis and transgenesis is explained by taking the national aspects into consideration.	Size of the ICC; Magnitude of Loadings of the factors at the national level

## Results

We report in Table 4 the comparison of DICs for the various models. This comparison (a huge difference of 553 between the *DICs* of models $M_{6}$ and $M_{5}$) leads to selection of the model $M_{6}$ (with two individual correlated factors and two national correlated factors) notably due to a considerable reduction of parameters in relation to $M_{5}$ even if $\bar{D}$ is slightly higher. Therefore, we report and comment only the model $M_{6}$ in the next tables.

**Table 4:** Comparison of DIC according to the different estimations.

	$\bar{D}$	*P*_*D*_	*DIC*
$M_{1}$ (1 individual factor)	198877.1	13324.5	212201.6
$M_{2}$ (1 individual factor and 1 national factor)	193719.9	13497.5	207217.4
$M_{3}$ (2 uncorrelated individual factors)	166037.3	23514.6	189551.9
$M_{4}$ (2 correlated individual factors)	166469.6	22486.8	188956.4
$M_{5}$ (2 uncorrelated individual factors 2 uncorrelated national factors)	162243.6	23558,1	185801.7
$M_{6}$ (2 correlated individual factors 2 correlated national factors)	162602.4	22645.7	185248.1

Note: $\bar{\text{D}}$: Average deviance; P_D_: Effective number of classes; DIC: Deviance Information Criterion.

Table 5 **explains what these different factors are made of.** On the first factor $\lambda_{1,r}^{\left(2\right)}$, the responses "unnatural", "harms environment", and "make me feel uneasy" are the best represented on the positive side. The responses "useful" and to be "encouraged" go the other way. This factor can be interpreted as a general attitude of opposition to cisgenesis at the national level. The factor $\lambda_{1,r}^{\left(1\right)}$ at the individual level can be interpreted in a similar manner even if we can see here that the standardized loadings are considerably higher. In other words, the individual determining factors have considerably more influence than the national determining factors for the attitude towards cisgenesis. For the factors$\lambda_{2,r}^{\left(2\right)}$ and $\lambda_{2,r}^{\left(1\right)}$, concerning transgenesis, the interpretation is similar except on one point. We can see that at both individual and national levels, the responses to the questions "promising idea", "safe", and "to be encouraged" go against the other responses. We can also compare the factors $\lambda_{1,r}^{\left(2\right)}$ and $\lambda_{2,r}^{\left(2\right)}$ for the questions asked in an identical manner for both types of gene transfer (namely: harms the environment, unnatural, makes me feel uneasy, to be encouraged, and GM label). With the exception of questions concerning the fact the gene transfers should be encouraged, we can see that the standardized loadings are greater for $\lambda_{1,r}^{\left(2\right)}$ than for $\lambda_{2,r}^{\left(2\right)}$. In short, the national context has more influence on the attitude towards cisgenesis than transgenesis.

**Table 5:** Estimation of loadings.

	Country factors						Individual factors					
	$\lambda_{1,\text{r}}^{\left(2\right)}$			$\lambda_{2,\text{r}}^{\left(2\right)}$			$\lambda_{1,\text{r}}^{\left(1\right)}$			$\lambda_{2,\text{r}}^{\left(1\right)}$		
	p.f.	s.e.	std.	p.f.	s.e.	std.	p.f.	s.e.	std.	p.f.	s.e.	std.
Gene transfer from other species												
Is a promising idea	0	0	0	-0.38	0.07	-0.25	0	0	0	-1.51	0.04	-0.99
Will be safe	0	0	0	-0.48	0.09	-0.30	0	0	0	-1.60	0.04	-1.01
Will harm the environment	0	0	0	0.29	0.07	0.23	0	0	0	0.91	0.02	0.74
Is fundamentally unnatural	0	0	0	0.18	0.09	0.14	0	0	0	0.96	0.02	0.75
Makes you feel uneasy	0	0	0	0.41	0.10	0.27	0	0	0	1.48	0.04	0.96
Should be encouraged	0	0	0	-0.51	0.09	-0.28	0	0	0	-2.05	0.07	-1.11
Is to be given a label	0	0	0	0.10	0.08	0.09	0	0	0	0.56	0.02	0.50
Gene transfer from the same species												
Will be useful	-0.24	0.08	-0.19	0	0	0	-1.08	0.02	-0.82	0	0	0
Will be risky	0.35	0.10	0.22	0	0	0	1.59	0.03	0.99	0	0	0
Will harm the environment	0.48	0.09	0.30	0	0	0	1.64	0.04	1.02	0	0	0
Is fundamentally unnatural	0.49	0.10	0.29	0	0	0	1.69	0.04	1.02	0	0	0
Makes you feel uneasy	0.73	0.13	0.32	0	0	0	2.70	0.08	1.18	0	0	0
Should be encouraged	-0.30	0.07	-0.21	0	0	0	-1.24	0.03	-0.89	0	0	0
Is to be given a label	0.24	0.07	0.20	0	0	0	0.70	0.02	0.60	0	0	0

Note: Posterior means are reported. p.f.: loading; s.e.: standard error; std.: standardized loading.

We have also $\sigma_{\text{n}12}^{\left(2\right)}=0.43\;\left(\text{standard error}=0.19\right)$ and $\sigma_{\text{n}12}^{\left(1\right)}=0.54\;\left(\text{standard error}=0.01\right)$. Both factors are strongly correlated at the national and individual levels (and slightly more at the individual level). These empirical findings are clearly in line with our first hypothesis (H1).

Table 6 **summarizes the importance of the inclusion of a "country level".** The inclusion of this level accounts for 3 to 8% of the total variance depending on the response. Interpretation of the ICC value differs among researchers, with some arguing that a value less than 5% indicates that multilevel modeling is not needed, whereas others advocate that even small amounts of variance can result in significant differences in model fit, in the presence of categorical variables [17, 76, 77]. Such values, although moderate in terms of latent responses, imply variations in the probabilities of responses observed for each country. This last point is confirmed by the loading in the previous table.

**Table 6:** Institutional influence explained by different levels.

	${\text{Var}}_{1}\left({\text{y}}_{\text{rij}}^{*}\right)$	${\text{Var}}_{2}\left({\text{y}}_{\text{rij}}^{*}\right)$	$\text{Var}\left({\text{y}}_{\text{rij}}^{*}\right)$	ICC	${\text{Com}}_{\text{r}}^{\left(1\right)}$	${\text{Com}}_{\text{r}}^{\left(2\right)}$	${\text{Com}}_{\text{r}}$	$\sigma_{\text{ur}}^{\left(2\right)}$
Gene transfer from other species								
Is a promising idea	2,24	0.07	2.31	0.03	0.54	0.03	0.56	0.01
Will be safe	2.39	0.12	2.51	0.05	0.55	0.04	0.59	0.02
Will harm the environment	1.45	0.08	1.54	0.06	0.30	0.02	0.32	0.05
Is fundamentally unnatural	1.50	0.13	1.63	0.08	0.31	0.01	0.31	0.12
Makes you feel uneasy	2.19	0.18	2.38	0.08	0.50	0.03	0.53	0.11
Should be encouraged	3.29	0.12	3.40	0.03	0.67	0.03	0.70	0.01
Is to be given a label	1.17	0.09	1.27	0.07	0.14	0.00	0.14	0.09
Gene transfer from the same species								
Will be useful	1.64	0.10	1.73	0.06	0.37	0.02	0.38	0.07
Will be risky	2.38	0.19	2.57	0.08	0.54	0.02	0.56	0.14
Will harm the environment	2.464	0.13	2.60	0.05	0.56	0.04	0.60	0.03
Is fundamentally unnatural	2.55	0.20	2.75	0.07	0.57	0.04	0.60	0.10
Makes you feel uneasy	4.97	0.27	5.24	0.05	0.76	0.04	0.80	0.04
Should be encouraged	1.84	0.09	1.93	0.05	0.43	0.02	0.45	0.06
Is to be given a label	1.27	0.09	1.36	0.07	0.20	0.02	0.21	0.07

Note: Posterior means are reported.

The response to the question whether transgenesis is “perceived unnatural", "make people feel uneasy" and "to be given a label" is more readily explained by the unobserved variables at the country level ($\sigma_{\text{ur}}^{\left(2\right)}$ is relatively high). They also have a total communality (*Com*_*r*_) that is relatively low (very low for the request for a label). They tend to vary independently of other responses. Here we can see a greater influence of the way in which public debate is structured. The response "a promising idea" or "to be encouraged" is less explained by the observable national variables (as $\sigma_{\text{ur}}^{\left(2\right)}$ is very low), whilst considering it as “unnatural” or that it leads to “feeling uneasy” depends considerably on unobservable variables. Concerning cisgenesis, the same interpretation holds concerning the responses “risky” or “unnatural”, which are highly dependent on unobservable national variables with a relatively high ICC. Feeling uneasy with this technique is, on the other hand, highly dependent on observable individual variables ($Com_{r}^{\left(1\right)}$ high and ICC low). In general, with higher *Com*_*r*_, the attitude regarding cisgenesis appears to be more homogenous than that of transgenesis. To resume, multilevel factor analysis gives us mixed evidence about the importance of national influence (H4).

**Lastly, the final table (**Table 7**) provides an understanding of the influence of different explanatory variables on the response to different questions from European reports on transgenic and cisgenic apples.** As these are "probit" responses, the estimation of the marginal effect is relatively straightforward. These marginal effects correspond to discrete changes for independent dichotomous variables [78]. We thus highlight the strong effects of age concerning attitudes to transgenic apples, whereas age seems to have less influence on the attitudes regarding cisgenic apples. Practicing a religion increases the probability of replying that transgenic apples harm the environment (+3%), are "unnatural" (+4%), and make people feel uneasy (+13%). This also leads to increasing the probability of replying that cisgenic apples are risky (+8%), harm the environment (+5%), and make people feel uneasy (+12%). Among other things, practicing a religion also reduces the probability of considering cisgenic apples as useful (-3%), and as to be encouraged (-3%). Lastly, in relation to our research hypotheses H2 and H3, we underline the fact that expressing an interest in the environment has contrasted effects on the respective acceptability of cisgenic apples (+9% as useful, +7% as unnatural, +4% to be encouraged, +4% to be given a label) and transgenic apples (+7% harm the environment, +12% unnatural, +17% make feel uneasy, +12% to be given a label). Conversely, concerning people with an interest in science or biotechnology, the effects are more similar between the two technologies leading to a greater acceptability. However, each time there is a positive effect on the demand for creating a label specific to these apples.

**Table 7:** Estimations^1^.

	Gene transfer from other species							Gene transfer from the same species						
	Promising idea	Safe	Harm envir.	Unnatural	Feel uneasy	Encourage.	Label	Useful	Risky	Harm envir.	Unnatural	Feel uneasy	Encourage.	label
Intercept	-0.723***	-1.42***	-0.64***	0.15	-0.55***	-1.76***	0.34***	-0.49***	-0.40**	-0.93***	0.18	-0.85***	-1.12***	0.50***
age25	-0.18**	-0.08	-0.08	0.07	0.10	-0.26**	0.09	-0.10	-0.08	-0.03	-0.04	0.17	0.01	0.03
m.e.^2^	-0.05	-0.01	-0.03	0.03	0.04	-0.02	0.03	-0.03	-0.03	-0.01	-0.02	0.05	0.00	0.01
age35	-0.28***	-0.27***	0.00	0.15**	0.26***	-0.34***	0.06	-0.06	-0.17*	-0.03	-0.17*	0.20	0.06	-0.02
m.e.	-0.08	-0.03	0.00	0.06	0.09	-0.02	0.02	-0.02	-0.06	-0.01	-0.07	0.06	0.01	-0.01
age45	-0.43***	-0.30***	-0.03	0.18**	0.41***	-0.51***	0.08	-0.10	-0.15	-0.14	-0.24**	0.16	0.04	-0.07
m.e.	-0.11	-0.04	-0.01	0.07	0.15	-0.03	0.03	-0.03	-0.05	-0.03	-0.10	0.05	0.01	-0.03
age55	-0.40***	-0.31***	-0.02	0.11	0.34***	-0.61***	0.05	-0.11	-0.28***	-0.14	-0.27***	0.17	0.03	-0.05
m.e.	-0.11	-0.04	-0.01	0.04	0.13	-0.03	0.02	-0.04	-0.10	-0.03	-0.11	0.05	0.01	-0.02
age65	-0.41***	-0.35***	-0.07	0.09	0.37***	-0.58***	-0.05	-0.11	-0.21*	-0.08	-0.25**	0.27	0.01	-0.17**
m.e.	-0.10	-0.04	-0.02	0.03	0.14	-0.03	-0.02	-0.04	-0.07	-0.02	-0.10	0.08	0.00	-0.06
Female	-0.36***	-0.38***	0.15***	0.15***	0.35***	-0.45***	0.10***	-0.16***	0.07*	0.06	0.11***	0.27***	-0.16***	0.00
m.e.	-0.10	-0.04	0.05	0.06	0.13	-0.03	0.04	-0.05	0.03	0.02	0.04	0.08	-0.03	0.00
Left	0.21***	0.20***	0.12***	0.14***	0.16***	0.19**	0.05	0.23***	0.11*	0.13**	0.03	0.09	0.25***	-0.02
m.e.	0.07	0.03	0.04	0.06	0.06	0.02	0.02	0.09	0.04	0.04	0.01	0.03	0.06	-0.01
Center	0.28***	0.24***	0.05	0.06	0.00	0.20***	0.09**	0.24***	0.09	0.07	0.01	-0.02	0.23***	0.06
m.e.	0.09	0.04	0.02	0.03	0.00	0.02	0.03	0.09	0.03	0.02	0.00	-0.01	0.06	0.02
Right	0.40***	0.43***	-0.02	0.01	-0.14**	0.45***	0.04	0.28***	0.03	-0.07	-0.03	-0.19**	0.34***	-0.05
m.e.	0.14	0.08	-0.01	0.00	-0.05	0.06	0.01	0.11	0.01	-0.02	-0.01	-0.05	0.09	-0.02
Familysize1	0.06	0.06	0.02	0.04	0.01	0.01	0.06	0.14***	0.04	-0.08	-0.06	-0.06	0.07	0.00
m.e.	0.02	0.01	0.01	0.01	0.00	0.00	0.02	0.05	0.02	-0.02	-0.02	-0.02	0.01	0.00
Familysize2	-0.04	0.01	-0.03	0.09*	0.07	-0.09	0.13***	0.07	0.01	-0.15**	0.02	-0.05	0.01	0.07
m.e.	-0.01	0.00	-0.01	0.03	0.03	-0.01	0.05	0.03	0.01	-0.04	0.01	-0.01	0.00	0.02
Familysize3	0.07	-0.02	0.00	0.07	0.02	-0.01	0.09*	0.08	-0.01	-0.09	0.02	-0.02	0.03	0.02
m.e.	0.02	-0.00	0.00	0.03	0.01	0.00	0.03	0.03	-0.01	-0.02	0.01	-0.00	0.01	0.01
edu16	0.01	-0.03	0.04	0.09**	0.09	0.01	0.08*	0.07	-0.12**	-0.12*	-0.03	0.05	0.08	0.02
m.e.	0.00	-0.00	0.01	0.04	0.03	0.00	0.03	0.03	-0.04	-0.03	-0.01	0.01	0.02	0.01
edu20	0.14**	0.05	0.02	0.01	-0.04	0.01	0.15***	0.16***	-0.26***	-0.16**	-0.09	-0.14	0.05	0.05
m.e.	0.05	0.01	0.01	0.00	-0.01	0.00	0.06	0.06	-0.09	-0.04	-0.03	-0.04	0.01	0.02
Stillstud	0.25**	0.13	-0.05	0.08	-0.23*	0.10	0.08	0.33***	-0.25*	-0.16	-0.18	-0.47***	0.22**	-0.09
m.e.	0.09	0.02	-0.02	0.03	-0.07	0.01	0.03	0.12	-0.09	-0.04	-0.07	-0.11	0.05	-0.03
sciencenat	0.20***	0.12**	-0.01	-0.02	-0.14***	0.20***	0.06*	0.22***	-0.10**	-0.08*	-0.11**	-0.37***	0.23***	-0.03
m.e.	0.06	0.02	-0.00	-0.01	-0.05	0.02	0.02	0.08	-0.04	-0.02	-0.05	-0.09	0.06	-0.01
Manager	0.03	0.05	-0.03	-0.06	-0.11	-0.08	0.06	0.10	-0.06	-0.09	-0.03	-0.25*	-0.03	-0.01
m.e.	0.01	0.01	-0.01	-0.02	-0.04	-0.01	0.02	0.04	-0.02	-0.02	-0.01	-0.06	-0.01	-0.00
Employee	0.02	0.00	-0.11	-0.01	-0.07	0.00	0.03	0.20**	-0.02	-0.10	-0.13	-0.32**	0.08	-0.02
m.e.	0.01	0.00	-0.03	-0.01	-0.02	0.00	0.01	0.07	-0.01	-0.03	-0.05	-0.08	0.02	-0.01
Worker	0.01	0.01	0.02	0.04	0.01	0.01	0.00	0.11	0.04	-0.03	-0.10	-0.20	0.09	-0.09
m.e.	0.00	0.00	0.01	0.01	0.00	0.00	0.00	0.04	0.01	-0.01	-0.04	-0.05	0.02	-0.03
Homeperson	-0.15	-0.01	-0.09	-0.12	-0.17*	-0.17	-0.03	-0.02	-0.16	-0.06	-0.03	0.04	-0.17*	-0.07
m.e.	-0.06	-0.00	-0.03	-0.05	-0.06	-0.01	-0.01	-0.01	-0.06	-0.02	-0.01	0.01	-0.03	-0.02
Unemployed	-0.10	-0.04	-0.05	-0.05	-0.01	-0.02	0.05	0.06	-0.07	-0.14	-0.15	-0.23	0.01	-0.06
m.e.	-0.03	-0.01	-0.02	-0.02	-0.01	-0.00	0.02	0.02	-0.03	-0.03	-0.06	-0.06	0.00	-0.02
Retired	-0.06	-0.06	-0.01	0.01	0.02	0.06	0.08	0.04	-0.06	-0.11	0.06	-0.09	0.03	-0.01
m.e.	-0.02	-0.01	-0.00	0.00	0.01	0.01	0.03	0.01	-0.02	-0.03	0.02	-0.02	0.01	-0.00
Smalltown	0.04	0.07	-0.03	-0.03	-0.05	0.09	-0.04	0.00	-0.06	-0.07	-0.06	-0.13*	0.05	-0.03
m.e.	0.01	0.01	-0.01	-0.01	-0.02	0.01	-0.01	0.00	-0.02	-0.02	-0.02	-0.04	0.01	-0.01
Bigtown	-0.04	0.01	-0.05	-0.07*	-0.07	0.02	-0.07*	-0.06	0.02	0.01	-0.04	0.01	-0.05	-0.01
m.e.	-0.01	0.00	-0.02	-0.03	-0.02	0.00	-0.03	-0.02	0.01	0.00	-0.02	0.00	-0.01	-0.00
Reliatt	-0.07	-0.02	0.13***	0.07*	0.34***	-0.10	0.00	-0.09*	0.20***	0.17***	**0.06**	0.39***	-0.15***	0.05
m.e.	-0.02	-0.00	0.04	0.03	0.13	-0.01	0.00	-0.03	0.08	0.05	0.03	0.12	-0.03	0.02
Relinonattend	0.02	0.06	0.05	0.04	0.24***	0.05	-0.02	0.02	0.12**	0.14***	0.10**	0.28***	-0.03	0.00
m.e.	0.01	0.01	0.02	0.02	0.09	0.00	-0.01	0.01	0.04	0.04	0.04	0.09	-0.01	0.00
interestenvir	0.01	-0.09	0.20***	0.32***	0.45***	-0.04	0.35***	0.25***	0.02	0.01	0.18***	0.13	0.17***	0.12***
m.e.	0.00	-0.01	0.07	0.12	0.17	-0.00	0.12	0.09	0.01	0.00	0.07	0.04	0.04	0.04
Interestscien	0.27***	0.30***	-0.01	-0.02	-0.11**	0.42***	0.10***	0.18***	0.01	0.01	-0.05	-0.15*	0.27***	0.11***
m.e.	0.09	0.05	-0.01	-0.01	-0.04	0.05	0.04	0.07	0.00	0.00	-0.02	-0.04	0.07	0.04
interestbiotec	0.38***	0.37***	0.27***	0.19***	0.26***	0.51***	0.22***	0.52***	0.00	0.04	-0.03	0.00	0.56***	0.06*
m.e.	0.13	0.07	0.10	0.07	0.10	0.07	0.08	0.20	0.00	0.01	-0.01	0.00	0.16	0.00

Note

^1^ Posterior means are reported.

^2^: m.e.: marginal effects.

*0 not included in the 90% credible interval;

** 0 not included in the 95% credible interval;

*** 0 not included in the 99% credible interval.

## Discussion and conclusion

We have highlighted a general attitude toward genetically modified apples. The two factors expressing opposition to transgenic and cisgenic apples are highly correlated at individual and national levels. In general with a higher *Com*_*r*_, the attitude toward cisgenesis appears to be more homogenous than toward transgenesis. For the latter, we find the same type of heterogeneity (plurality of attitudes, justification means, types of opposition) as defined in previous publications [4, 34, 39, 79]. In coherence with other studies on European consumers [18, 38], we underline opposition concerning all genetic engineering techniques even if our study reveals mixed responses with contrasted impacts of being interested in the environment. It is as if cisgenic apples have become part of a new "utility"/"risk" dilemma as highlighted previously by Gaskell *et al*. [34]. In effect, we underline a more important age effect for cisgenesis than for transgenesis, with increasingly weaker support as age increase (as in Rousselière & Rousselière [47]. Consumers can balance out the risk with expected benefits for technology, but this connection is plural [34], depending on the social and cognitive resources available that may influence their perception of biotechnologies. Note however that the effect of population ageing may be complex and can have structural effects on European societies. The development of functional foods or organic food even with new biotechnologies for example may lead to a greater acceptance by middle-aged and elderly consumers [13, 80].

Contrary to previous research, our empirical strategy allows us to test simultaneously four hypotheses (see Table 8). Our first hypothesis H1 seems therefore validated as a high correlation between social acceptability of cisgenesis and transgenesis had been highlighted, with a higher acceptance of the latter. In relation to our research hypotheses H2 and H3, we underline the fact that expressing an interest in the environment has contrasted effects on the acceptability of cisgenic and transgenic apples. Conversely, expressing an interest in science or biotechnology leads to greater acceptability. Finally, our multilevel modeling provides mixed evidence about H4. Although factor loadings are significant at the national level, the estimated values of the various ICCs seem relatively low, or at least mild according to various rules of thumb. Therefore, it is as if there is a convergence between European countries. Unfortunately our model is not flexible enough to include random effects, as in Rousselière & Rousselière [47] where divergence between European countries is largely explain by the strategy of national political parties. However, if we compare transgenesis and cisgenesis, there is still a high difference between countries about social acceptability. Although our work is nonetheless an extension of previous research, one way to address these issues is to extend our work to finite mixture modeling. Multilevel Latent class can allow us to provide a typology of individuals that can be useful to understand simultaneously the various profiles of opponents to biotechnologies and the typology of countries [81, 82]. New developments (mixture structural equation models) proposed by Lee & Song [83] that allow parameters to vary for various cluster may be a fruitful modeling for future studies.

**Table 8:** Validation of the hypothesis.

Hypothesis	Validation
H1. There is a greater acceptability of cisgenesis than transgenesis, while noting a strong correlation between the two.	Yes
H2. Environmental concerns are important for the acceptability of both techniques	Yes (in part). Marginal effects of Interestenvir significant for 8 responses
H3. General interest in science or biotechnology are important factors influencing the acceptability of both techniques.	Yes (in part). Marginal effects of interestscience and interestbiotech significant for respectively 9 and 10 responses.
H4. A considerable portion of the heterogeneity of individual preference towards cisgenesis and transgenesis is explained by taking the national aspects into consideration.	Mixed evidence: low ICC (between 3 and 8%) but significant loadings at the national factors

Several issues can be emphasized in closing. The first concerns public policy toward biotechnologies. The study confirms the presence of clear differences in the fields of application for biotechnologies. The different studies carried out on the social acceptability of biotechnologies highlight that the absence of perceived utility is a key point [1, 32, 33, 84, 85]. Medical treatments developed from biotechnologies are considered less risky than the development of an illness. Inversely, the development of biotechnologies in ornamental horticulture, in other words, the use of biotechnologies in an explicitly leisure context, is strongly rejected [84, 86, 87].

The second issue concerns the differences observed between different European countries. Our article highlights a greater variability in attitudes toward cisgenesis between European countries in comparison with transgenesis. Significantly, this is also the result found by Lusk & Rozan [51] when they compared the United States and France. Thus, according to these authors, intraspecies transfers or intragenesis transfers are mainly accepted by American consumers (between 52.7% for the transfer of numerous genes of different plants to 77.3% for the transfer of a gene coming from the same plant) while they are mainly refused by French consumers (respectively 17.5% to 37.5% support). Consumers in both countries reject other types of gene transfers overall. This study could be extended to understand the origins of this difference of opinion between countries. According to different studies, the acceptability difference first stems from a "trust gap" between countries highlighted by Priest *et al*. [88]. While controlling the level of knowledge, trust in scientists [89, 90], public authorities [91, 92] or manufacturers [5] have a positive impact on the acceptability of genetically modified foods, distrust in public authorities leads to a greater acceptance of alternative foods (organic or local) [93]. On the other hand, trust in environmental associations [94, 95] reduces its acceptability. The “trust gap” explains the difference in acceptability of GMOs in Europe and in the United States by the fact that Europeans have a greater trust in consumer and environmental protection associations, and in the United States people have a greater trust in the "biotechnology system".

This study confirms that it is necessary to distinguish between an increase in the flexibility of regulations regarding organisms arising from cisgenesis (relative to regulations for organisms coming from transgenesis), and an absence of product labeling policies for these organisms. Advocates of cisgenesis recognize this distinction [8, 96]. If cisgenesis is likely to encounter greater acceptability among European consumers, there is still considerable opposition to contend with (beyond the question of breaking through the "barrier between species" or the environmental argument). On the contrary, we know that consumer tolerance to apple scab is possible with a label indicating organic agriculture and/or on more environmentally friendly practices [97, 98]. We also find elements in support of the position of the European Commission that classifies this type of technique as particularly close to more traditional transgenic techniques [30]. The creation of a label for this type of product is requested if these types of products were to be developed and authorized for sale. Nonetheless, as for all species subject to pollen dispersion, the question of coexistence between different techniques remains [99, 100].

## Supporting information

S1 Appendix Descriptive statistics. (DOCX)
