# Supplementary material for: Is biotechnology (more) acceptable when it enables a reduction in phytosanitary treatments? A European comparison of the acceptability of transgenesis and cisgenesis
Source: PLoS One. 2017 Sep 6;12(9):e0183213. doi: 10.1371/journal.pone.0183213 (PMC5587272; doi:10.1371/journal.pone.0183213)
Supplement: S1 Appendix — (DOCX) [file pone.0183213.s001.docx]

**Appendix 1. Descriptive statistics**

| **Variable** | **Label** | **Min** | **Max** | **Mean** | **Standard error** |
| --- | --- | --- | --- | --- | --- |
| AGE15 | Age : from 15 to 24 years old | 0 | 1 | 0.13 | 0.33 |
| AGE25 | Age : from 25 to 34 | 0 | 1 | 0.16 | 0.37 |
| AGE35 | Age: from 35 to 44 | 0 | 1 | 0.17 | 0.38 |
| AGE45 | Age from 45 to 54 | 0 | 1 | 0.17 | 0.37 |
| AGE55 | Age from 55 to 64 | 0 | 1 | 0.16 | 0.37 |
| AGE65 | Age : over 64 | 0 | 1 | 0.21 | 0.41 |
| Female | Gender: Female | 0 | 1 | 0.53 | 0.50 |
| Left | Political scale: left | 0 | 1 | 0.25 | 0.43 |
| Center | Political scale: center | 0 | 1 | 0.31 | 0.46 |
| Right | Political scale: right | 0 | 1 | 0.22 | 0.41 |
| No_opinion | Political scale: don’t know or refusal | 0 | 1 | 0.13 | 0.34 |
| Family_size1 | Family : one person | 0 | 1 | 0.21 | 0.40 |
| Family_size2 | Family : two people | 0 | 1 | 0.33 | 0.47 |
| Family_size3 | Family: three people | 0 | 1 | 0.19 | 0.39 |
| Family_size4+ | Family : four people and more | 0 | 1 | 0.28 | 0.45 |
| Education15- | Stop full-time education: 15 years old or less | 0 | 1 | 0.18 | 0.38 |
| education16_19 | Stop full-time education: 16 to 19 | 0 | 1 | 0.41 | 0.49 |
| education 20+ | Stop full-time education: 20 years old and more | 0 | 1 | 0.30 | 0.46 |
| Student | Stop full-time education: Still Studying | 0 | 1 | 0.09 | 0.29 |
| Natural_science | studied natural science, technology or engineering: at school, in college, in the university or anywhere else. | 0 | 1 | 0.44 | 0.50 |
| Self_employed | Occupation: Self_employed | 0 | 1 | 0.07 | 0.26 |
| Manager | Occupation: Manager | 0 | 1 | 0.11 | 0.31 |
| Employed_position | Occupation: Employed_position | 0 | 1 | 0.11 | 0.31 |
| Manual_worker | Occupation: Manual_worker | 0 | 1 | 0.19 | 0.39 |
| Homemaker | Occupation: Homemaker | 0 | 1 | 0.08 | 0.27 |
| Unemployed | Occupation: Unemployed | 0 | 1 | 0.08 | 0.27 |
| Retired | Occupation: Retired | 0 | 1 | 0.27 | 0.44 |
| Rural | Live in : Rural area or village | 0 | 1 | 0.35 | 0.48 |
| Smalmiddleltown | Live in: Small or middle sized town | 0 | 1 | 0.36 | 0.48 |
| Bigtown | Live in : Large town | 0 | 1 | 0.28 | 0.45 |
| noreli | Non-religious | 0 | 1 | 0.25 | 0.44 |
| Relinonattend | Religious without attending religious services | 0 | 1 | 0.42 | 0.49 |
| Reliattend | Religious and attending religious services | 0 | 1 | 0.33 | 0.47 |
| Interestenviro | Interested in environment | 0 | 1 | 0.88 | 0.33 |
| Interestscience | Interested in science | 0 | 1 | 0.77 | 0.42 |
| Interestbiotech | Interested in biotechnology | 0 | 1 | 0.73 | 0.44 |
